# The utility of a multimedia education program for prostate cancer patients: a formative evaluation

**DOI:** 10.1038/sj.bjc.6602071

**Published:** 2004-08-03

**Authors:** D Flynn, P van Schaik, A van Wersch, T Ahmed, D Chadwick

**Affiliations:** 1 School of Social Sciences and Law, University of Teesside, Middlesbrough TS1 3BA, UK; 2 Department of Urology, James Cook University Hospital, Middlesbrough TS4 3BW, UK

**Keywords:** prostate cancer, knowledge, psychosocial functioning, decision-making, multimedia, information needs

## Abstract

A multimedia program (MMP) was developed to educate patients with prostate cancer about their disease. A within-subjects design was used to investigate the changes in levels of cancer-related knowledge, psychosocial functioning, treatment decision-making role and information needs immediately after browsing the MMP. The participants were 67 men recently diagnosed with prostate cancer. Psychosocial functioning was assessed with 20 items describing common emotional states and coping strategies employed by cancer patients. Treatment decision-making role was assessed with the Control Preference Scale. A principle component analysis of the 20 psychosocial items yielded three components: distress, positive approach and nonacceptance. After browsing the MMP significant increases in knowledge and reductions in distress were reported. Marital status was significantly associated with knowledge gain. Married men and those attending the study session with their spouse displayed a significant shift towards a more active role in treatment decisions. The majority of information needs were fulfilled by the MMP; however, information related to the likelihood of a cure, treatment side effects, coping strategies and aetiology were not completely satisfied by the MMP. Implications of the findings and suggestions for future work on the design and evaluation of the MMP are discussed.

## 

In the UK, one in 14 men are at a lifetime risk of developing prostate cancer, which is the second leading cause of death in men after lung cancer, accounting for 9280 deaths in 2000 (Cancer Research UK, 2002a, 2002b). Treatments for prostate cancer include surgery, hormone therapy, radiotherapy, chemotherapy and active monitoring (or watchful waiting). Treatment side effects are numerous and can occur for short periods, whereas others such as incontinence and impotence have long-term effects that impact negatively upon quality of life. However, the probability of experiencing side effects associated with particular treatments is unclear from the literature, with large differences in frequency, duration and severity between studies. Furthermore, the relative survival benefit of different treatments has yet to be elucidated; consequently, there is still no unequivocal evidence to support one treatment over another (Holmberg *et al*, 2002).

### Psychosocial functioning

Psychosocial problems experienced by men with cancer have received sparse attention compared to women in the research literature. This difference in attention is hard to justify as research has shown that men with prostate cancer experience psychosocial problems such as social role changes, financial worries, anger, depression and anxiety regarding treatment and potential death (Gregoire *et al*, 1997; Gray *et al*, 1999). Research has also reported that men with cancer rarely seek help for psychosocial problems (Jorm, 1994); have little awareness of coping strategies (Whitrod, 1996); are often denied information on positive coping by clinicians (Fitch *et al*, 1999); and are prone to relying upon avoidance-coping strategies associated with poor psychological outcomes and decreased survival rates (Shrock *et al*, 1999). Furthermore, men appear to receive little emotional support other than from their spouse (Helgason *et al*, 2001) who also experience psychosocial problems in response to their partner's diagnosis (Gray *et al*, 1999).

### Patient education

Patient education (information provision) has been proven to be an effective strategy for alleviating psychosocial problems in both men and women with cancer (Fallowfield *et al*, 1997; Davison *et al*, 2003). Information provides a sense of control, reduces distress, facilitates adaptive coping, and increases participation in shared decision-making (SDM) with physicians (Gregoire *et al*, 1997; van Wersch *et al*, 1997a, 1997b; Davison *et al*, 2003). Effective information provision should enrich doctor–patient interactions by transforming consultations into negotiations between expert patients and expert physicians; however, in reality it is clinicians, not patients, who are in possession of the knowledge required to make an informed decision (Crawford *et al*, 1997). Despite the general agreement that men should be involved in treatment decisions, the type and amount of information needed for SDM has failed to reach a consensus (Feldman-Stewart *et al*, 1998). Davison *et al* (1995) reported the following hierarchical structure of information needs of men recently diagnosed with prostate cancer: likelihood of cure, stage of disease, available treatments, side effects on usual social activity, self-care, treatment side effects, hereditary risks, effects upon family and friends, and treatment effects upon sexual activity. Verbal consultations may fulfil these information needs but this information is subject to poor recall and understanding by patients (Michie *et al*, 1997). Consequently, patients are increasingly given printed information to reinforce, or in many cases to replace, verbal information provided by clinicians (Frank-Stromborg and Cohen, 1991). Currently, a combination of information provision (verbal and printed) with support from healthcare professionals is considered ‘good clinical practice’. This enables the healthcare professional to respond according to an individual patient's information needs.

The main disadvantages of printed information (and other media such as audio and videotapes) are that reading level is often inappropriate; these media possess limited information for patients who wish to pursue a deeper understanding; they are unable to adapt quickly to new information; salient topics are often missing; uncertainties are ignored; and they fail to provide a balanced account of the effectiveness of available treatments (Smith and Timoney, 1997; Coulter *et al*, 1999).

### Multimedia patient education

A multimedia program (MMP) is a computer-based application that combines text, sound, graphics, video and interactivity, which serve to reinforce and complement one another to facilitate learning. Multimedia programs presently offer the most comprehensive method of information provision that address several of the shortcomings associated with other media such as printed information. Multimedia programs can be easily and quickly updated to incorporate new treatment approaches and evidence from clinical trails that may refute previous information. Interactivity can provide autonomy as it enables patients to dictate the pace, type and the order information is viewed in the MMP, which enables more knowledgeable patients to access salient information more quickly without attending to previously accessed information. Multimedia programs provide all the benefits of patient education without increasing staff costs or time, and are capable of being accessed at home via the Internet or CD-ROM. Disadvantages of MMPs are initial development and start-up costs and technology acceptance by clinicians and patients.

Clinical trials of MMPs as health education tools have reported positive results for increasing patients' knowledge, information-seeking and participation in SDM (Krishna *et al*, 1997). Multimedia programs for patients with benign prostatic hyperplasia have reported positive results for reducing self-assessed prostate symptoms (van Schaik *et al*, 1999) and facilitating SDM (Barry *et al*, 1995; Shepperd *et al*, 1995; Wagner *et al*, 1995; Murray *et al*, 2001). Pilot studies of MMPs for prostate cancer have reported patient satisfaction with navigability, layout and content (Jenkinson *et al*, 1998; Brink *et al*, 2000). Patient outcomes such as participation in SDM were neglected, although Brink *et al* (2000) reported increased knowledge of cancer staging and brachytherapy (the only treatment that was included in the MMP) including increased patient self-efficacy for discussing brachytherapy with physicians. These studies demonstrated that MMPs can be effective media for increasing knowledge of the entire spectrum of treatment options for prostate cancer.

Therefore, the objective of the current study was to conduct a ‘formative evaluation’ by investigating the effect of the MMP on knowledge acquisition, psychosocial functioning, preference for participating in treatment decisions and information needs of patients recently diagnosed with prostate cancer. A formative evaluation is an evaluation that takes place before actual implementation of a final product, and which influences the development of the product (Preece *et al*, 1994). The results of the formative evaluation will be used to conduct a future ‘summative evaluation’ (undertaken after implementation of the final product, with the aim of testing the functioning of a product) of the final version of the MMP. Involving patients in the formative evaluation is in line with guidelines for conducting research affecting patients in the UK (DoH, 2001).

## MATERIALS AND METHODS

### Design

A within-subjects design was used to evaluate the utility of the MMP. The independent variables were study condition (pretrial – immediately before using the MMP and post-trial – immediately after using the MMP), patient age, education, living circumstances and employment status. The outcome measures were the level of cancer-related knowledge, psychosocial functioning, treatment decision-making role and information needs.

### Participants

The participants were 67 men recently diagnosed (1 week or less) with prostate cancer. The men were selected based on consultant urologists' assessment of their suitability for inclusion in the study. The age range was 48–89 with a mean age of 65.7 years (SD=7.95). The percentage of participants with secondary (school, aged ⩽16), further (college, aged ⩾16) and higher education (university, ages ⩾18) was 50, 36 and 14% respectively. The majority were married (90%), retired (76%), resided in their own homes with at least one other person (84%) and attended the study session with their spouse (70%).

### Multimedia program

An MMP was developed using previous research on the information needs of prostate cancer patients (e.g. Davison *et al*, 1995) and a working committee consisting of two consultant urologists, a health psychologist, a psychologist specialising in human–computer interaction and a multimedia developer. The MMP combined text with sound, narration, images, animation and streaming video. The MMP was comprised of six cancer-related modules: (a) prostate anatomy, (b) disease stages, aetiology and symptoms, (c) diagnostic techniques, (d) treatment options (surgery, hormone therapy and radiotherapy) and side effects, which included a research update, (e) coping strategies and (f) further information (self-help groups, prostate cancer organisations, further reading and a cancer glossary). The MMP was operated on a stand-alone PC and participants navigated through the MMP using a mouse. The interface used a selection of on-screen buttons (forward, back, exit) that controlled interaction and navigation through the MMP. Participants were instructed how to use the MMP by a research assistant who was present throughout the study session.

### Study questionnaire

A questionnaire in hard-copy format was used to assess psychosocial functioning, cancer-related knowledge, treatment decision-making roles and information needs. The first part of the questionnaire described the aims and objectives of the research and requested demographical information from the participants.

Cancer-related knowledge was assessed using 20 statements that were representative of the information presented in the six cancer-related modules of the MMP: cancer in general and prostate anatomy; disease advancement; and aims and side effects of surgery, radiotherapy and hormone therapy (see Table 1

**Table 1:** The 20 statements used to assess cancer-related knowledge

	**Knowledge**	
	**Pretrial**	**Post-trial**
*Cancer in general and prostate anatomy*	2.49^a^ (1.01)^b^	2.82 (0.75)
Male hormones are produced by the brain		
Cancer is a type of infection of tissue		
The prostate is part of the penis		
The prostate surrounds the first part of the tube which carries urine from the bladder to the penis		
		
*Disease advancement*	2.75 (1.29)	3.13 (1.08)
Cancer is a lump of cells that may invade and destroy surrounding tissues		
Prostate cancer can spread to other parts of the body		
Prostate cancer never spreads outside the prostate		
Growth of prostate cancer is driven by the male hormones		
		
*Aims and side effects of surgery*	3.07 (0.98)	3.22 (0.94)
The aim of prostate surgery is to remove part or all of the tumour in the prostate		
The aim of prostate surgery is to remove the testicles		
Possible side effects of prostate surgery include problems in control of the bladder		
After prostate surgery most prostate patients are incontinent		
		
*Aims and side effects of radiotherapy*	2.39 (1.05)	2.67 (0.68)
A possible side effect of radiotherapy is breast enlargement		
Possible side ffects of radiotherapy include tiredness and nausea		
The aim of radiotherapy is to destroy cancer cells while doing as little harm as possible to normal cells		
The aim of radiotherapy is to remove the prostate		
		
*Aims and side effects of hormone therapy*	1.92 (1.27)	2.55 (1.00)
The aim of hormone therapy is to increase the amount of male hormones		
Possible side effects of hormone therapy include the inability to have an erection		
The aim of hormone therapy is to slow down or shrink the tumour		
A possible side effect of hormone therapy is increased body strength		
		
Overall	12.62 (3.90)	14.38 (3.00)

a Mean.

b s.d.

). Participants responded to each statement as ‘true’, ‘false’ or ‘don't know’.

Previous research on emotional states and coping strategies employed by cancer patients was used to design a 20-item checklist to assess psychosocial functioning. Six items used adjectives as descriptors for internal emotional states (e.g. angry); the remaining 14 items used brief statements related to coping strategies for dealing with cancer (see Table 2

**Table 2:** Percentage and cumulative percentage of variance explained per component, and component loading matrix from the principle components factor analysis of the psychosocial functioning items

	**Component**		
**Item**	**Distress**	**Positive approach**	**Nonacceptance**
Shocked	0.77		
I cannot believe this has happened to me	0.53		
I expected it			
Angry			
Anxious	0.92		
Frightened	0.79		
Uncertain			
Blame myself			0.84
Miserable			
Having a good cry			
Running away			0.94
Pray to God			
Talk to other patient			
Talking to someone I trust		0.52	
I feel like going to another doctor to make sure it is true			0.74
Finding out more on prostate cancer			
Do not think about it			
Carry on with your life		0.75	
Fight this disease		0.74	
Enjoy myself as much as I can		0.86	
			
% age of variance explained	24.7	12.6	9.7
			
Cumulative %	24.7	37.3	47.0

Extraction method: principle components analysis. Rotation method: direct Oblimin with Kaiser normalisation. The stem question preceding the 20 psychosocial functioning items was ‘please tick one box for each of the following statements to indicate how you feel now about your prostate cancer’ (very much, a little or not at all).

). The participants responded to each item on a three-point Likert scale (not at all, a little and very much).

The Control Preference Scale developed by Degner and Sloan (1992) was used to assess the treatment decision-making role, which has been used previously to assess treatment decision-making preferences of prostate cancer patients (e.g. Davison *et al*, 1995). Participants indicated their preferred role in treatment decision-making (active, passive or collaborative) by selecting the category indicative of their status.

Information needs were assessed with free-response items that asked participants to state their most important information need at pre- and post-trial. Participants were also asked to state the most important knowledge they had acquired at post-trial.

### Procedure

Ethical approval for the study was granted by the Trust on the basis that it was part of existing practice to provide practical advice, guidance and support to men with newly diagnosed with prostate cancer. After the ‘bad news’ consultation the urologist or prostate cancer nurse informed the participant about the study and provided them with a study information sheet (that detailed the study aims and rationale) and a consent form. Participants were given the choice of participating immediately, or within 1 week after the initial bad news consultation. They were also given the choice of attending the study session with a significant other or alone. A private room situated within the urology department was used to conduct the study. Prior to browsing the MMP, the participants were asked to complete the study questionnaire, which was followed by instructions on how to use the MMP. No time limit was imposed on patients for browsing the MMP. After browsing the MMP, participants were requested to complete the study questionnaire for a second time. They were then fully debriefed and thanked for their time, cooperation and patience.

## RESULTS

### Knowledge acquisition

Overall numbers of correct responses to the 20 knowledge items increased between the pre- and post-trial conditions (see Table 1). A related *t*-test revealed that overall levels of correct responses significantly increased between the pre- and post-trial conditions (*t* [59]=4.49, *P*<0.001). A multiple regression analysis showed that being married was a significant predictor of overall knowledge gain between the pre- and post-trial conditions (*β*=0.31, *R*^2^=0.10, *P*<0.05).

Correct responses for each of the five knowledge domains increased between the pre- and post-trial conditions (see Table 1). A two-factor repeated measures ANOVA revealed a significant main effect of knowledge domain (F [3.5, 206.6]=15.67, *P*<0.001, MS_knowledge domain_=12.30, Greenhouse–Geisser correction applied) and a significant interaction effect between knowledge domain and study condition (F [3.19, 187.89]=12.22, *P*<0.001, MS_interaction_=12.91). A series of simple effect tests revealed that the following knowledge domains increased significantly between the pre- and post-trial conditions: cancer in general and prostate anatomy (*t* [59]=−2.34, *P*<0.05), disease advancement (*t* [59]=−2.92, *P*<0.01), aims and side effects of radiotherapy (*t* [59]=−2.25, *P*<0.05) and hormone therapy (*t* [59]=−4.51, *P*<0.001). Knowledge gain for aims and side effects of surgery failed to reach significance.

### Psychosocial functioning

Responses to the 20 psychosocial functioning items at pretrial were subjected to a principle components analysis. A three-component solution was extracted that explained 47% of the variance (see Table 2). Each component possessed adequate factor loadings (0.52–0.94) and internal reliability with Cronbach's alpha values of ⩾0.68. The three components were subsequently named distress (feelings of shock and fear), positive approach (optimism and a fighting spirit) and nonacceptance (denial) that explained 25, 13 and 10% of the variance respectively. Distress was significantly positively associated with both positive approach (*r* [61]=0.32, *P*<0.05) and nonacceptance (*r* [60]=0.31, *P*<0.05) in both study conditions. A related *t*-test revealed that distress decreased significantly between the pre- and post-trial conditions (*t* [58]=2.35, *P*<0.05).

### Treatment decision-making

In the pre- and post-trial study conditions, 68 and 71% respectively of participants preferred an active or collaborative role in treatment decisions. Wilcoxon tests revealed no significant differences in treatment decision-making roles between the pre- and post-trial study conditions; however, a significant shift in preferences for a more active role in treatment decisions was reported for (a) participants who attended the study session with their spouse or partner (*z* [42]=−2.49, *P*<0.05) and (b) participants who were married (*z* [47]=−1.98, *P*<0.05).

### Information needs

The information needs reported by the study participants were examined for common themes and coded into mutually exclusive categories (see Table 3

**Table 3:** Information needs at pre- and post-trial and most important knowledge acquired

	**Most important information need**		**Most important information still required**		**Most important knowledge acquired**	
	**Pretrial**		**Post-trial**		**Post-trial**	
	**F**	**% F**	**F**	**% F**	**F**	**% F**
Likelihood of a cure	19	28	10	15	3	4
Treatment side effects	10	15	4	6	1	2
Coping strategies	9	13	4	6	1	2
Diagnostic tests	8	12	0	0	1	2
Treatment duration	5	7	1	2	0	0
Aetiology	3	4	4	6	16	24
Hereditary risks	0	0	0	0	20	30
Disease advancement	0	0	0	0	3	4
Cannot decide	13	19	44	66	22	33
Totals	67	100	67	100	67	100

*Note*: Percentages may not equal 100 due to rounding.

). A frequency analysis revealed six categories of primary information needs at pretrial with the following hierarchical structure: likelihood of a cure (28%), treatment side effects (15%), coping strategies (13%), diagnostic tests (12%), treatment duration (7%) and aetiology (4%). In total, 19% stated that they had no information needs at pretrial.

At post-trial, five categories of information needs displayed at least a 40% decrease, with only aetiology displaying a negligible increase. Approximately 66% of the participants indicated that they required no further information needs at post-trial. Seven categories were reported as the most important knowledge acquired with the following hierarchical structure: hereditary risks (30%), aetiology (24%), likelihood of a cure (4%), disease advancement (4%), coping strategies (2%), diagnostic tests (2%) and treatment side effects (2%). Approximately 33% could not decide upon the most important knowledge they had acquired.

## DISCUSSION

Consistent with previous research evaluating patient education tools, patients in the current study reported significantly less distress (Gregoire *et al*, 1997; Davison *et al*, 2003), more cancer-related knowledge (Glajchen and Moul, 1996), and a desire for a more active role in treatment decisions (if they attended the session with their spouse/partner or were married) immediately after browsing the MMP.

The current study also demonstrated that men expressed similar patterns of psychosocial problems as women with cancer as they reported anxiety, fear and shock (distress) and utilised both positive (positive approach) and negative (nonacceptance) coping strategies (Fallowfield *et al*, 1997; van Wersch *et al*, 1997a, 1997b). The association between distress and nonacceptance was also consistent with previous research that found men with cancer are prone to relying upon avoidance coping strategies in response to the stress of a life-threatening disease such as cancer (Vingerhoets and Van Heck, 1990). The reduction in distress is an important finding given that less distressed patients are better able to make sense of their experience with cancer and seek desired information (Leydon *et al*, 2000).

The results of the current study were inconsistent with previous research using the Control Preference Scale (CPS) that reported the majority of men within 0–13 weeks of receiving their diagnosis preferred a passive decision-making role (Davison *et al*, 1995). However, more recent studies utilising the CPS are congruent with the current study reporting that 68% (Davison and Degner, 1997), 75% (Wong *et al*, 2000) and as many as 93% (Davison *et al*, 2002) of men recently diagnosed prefer either an active or collaborative role in treatment decisions. This trend in the current study could be attributed to the relatively low mean age of the study participants (Davison *et al*, 2002), and/or spousal support that served as a catalyst to learn and take part in shared decision-making (Ptacek *et al*, 1999).

The MMP adequately fulfilled information needs for treatment side effects, coping strategies, diagnostic tests and treatment duration. The failure of the MMP to completely satisfy information needs related to the likelihood of a cure and aetiology is probably attributable to the state of medical knowledge and the lack of prospective clinical trials of sufficient quality comparing one treatment with another rather than shortcomings of the MMP.

Browsing the MMP developed a need to acquire information other than those anticipated at pretrial and to reprioritise information needs. However, the hierarchical structure of information needs was inconsistent with previous research (Davison *et al*, 1995, 2002). The discrepancies with previous research may be due to using free-response questions, only requesting primary information needs, and 19% of men in the pretrial condition indicating they did not have any information needs, which increased to 66% at post-trial.

### Limitations

Despite these encouraging results, there are several methodological issues that may have had a confounding influence upon the outcome measures. The sampling method (selection by urologist) employed to select participants may have produced an unrepresentative sample as reasons for noninclusion were not recorded. Other methodological issues that potentially reduce generalisability of the results include the failure to record the participants' disease stage and functional status. Furthermore, given the generally late onset of prostate cancer, the mean age (66 years) of the study participants was relatively young.

The psychosocial functioning scale utilised in the current study needs to be further validated in future research, as only internal reliability and internal validity was assessed. A research assistant also supported the participants throughout the study session, which may have impacted upon the participants' level of distress. Furthermore, the question of whether the level of support provided is necessary in future applications of the MMP needs to be investigated.

### Suggested improvements to the MMP

The inclusion of a decision-aid to communicate quantitative outcome information to patients could address the shortcomings of the MMP in terms of fulfilling information needs. However, the decision-aid used to present outcome data must be processed with a high degree of accuracy by patients, otherwise it can influence the perception of probability (Chatterton, 1999). According to Feldman-Stewart *et al* (2000) a 10 × 10 matrix of shaded ovals is the most efficacious format for presenting information on probabilities to patients, although this must be explained with support from clinicians.

Coping knowledge may be enhanced by amalgamating information on positive coping strategies with other salient topics such as likelihood of cure. To enable men to vicariously learn positive coping strategies, streaming video of a real-patient (or trained actor) describing the probability of a cure associated with each disease stage could be followed by a description of how to develop positive coping strategies to deal with treatment side effects.

An assessment of reading level required to comprehend the information presented in the MMP needs to be conducted to ensure understanding by all patients irrespective of educational background. A self-test at the end of each topic that provides feedback on performance (and delivering reassurance and support in the case of poor performance) could also enhance learning via the use of positive reinforcement (operant conditioning). The inclusion of a search function would also facilitate learning as patients could pinpoint salient information needs more quickly and avoid the frustration of being unable to locate desired information. Following a summative evaluation, algorithms built into the MMP could *suggest* an appropriate treatment modality based on a patient's unique status (clinical profile and preferences regarding both positive and negative treatment outcomes).

### Future research

The use of the MMP for prostate cancer patients at this stage of evaluation cannot be recommended until prospective randomised control trials to compare the utility of the MMP with good clinical practice have been completed. In a summative evaluation, important factors such as ‘usability’ may have influenced the outcome measures in the current study. Usability refers to the ease of use and acceptability of a product for particular types of user to perform specific tasks in a given context, which is influenced by cost, convenience, availability, prerequisite training and organisational issues (Bevan and McLeod, 1994). Therefore, a combination of performance measures and assessments of user satisfaction is required to determine the usability of the MMP in both clinical and residential environments as a function of style and properties of the interface (e.g. methods used to communicate between the user and computer), dialogue structure, functionality (e.g. browsing content), efficiency (e.g. navigation structure), reliability (e.g. fault tolerance), user characteristics (e.g. age) including the combination of attributes that provide the greatest level of satisfaction for the majority of users. Particular attention should be given to obtaining information from patients who dislike or feel uncomfortable using computers. Ergonomic factors such as postural demands may also be related to perceived ease of use, especially in elderly men, and deserve consideration in future applications of this MMP.

High usability of the MMP is essential if the MMP is to be used in patients' homes or accessed via the WWW, to ensure that the MMP can successfully compete with the perennial increase in the number of cancer-related Web sites on the Internet. In particular, it must be established if the MMP confers benefits over time in terms of outcomes assessed in the current study, including satisfaction with care, quality of life and ultimately survival. Furthermore, future work should be conducted to determine if the MMP can fulfil post-treatment information needs and those of partner-caregivers who are reported to have information needs equivalent to those of patients (Davison *et al*, 2002).

## CONCLUSION

The incidence of prostate cancer is expected to increase in developed countries due to ageing populations, increased use of PSA screening and declines in other major causes of mortality. This will result in concomitant cost increases to health care providers and it is unlikely that the slow and expensive process of training biomedical practitioners occupying the central role in health care will meet the increased demand for their ‘expert knowledge’ (Weed, 1997). However, empowered with sufficient knowledge patients can make informed decisions about their treatment in collaboration with clinicians without an investment of staff time to deliver the information. Multimedia programs are unlikely to replace the ‘human touch’ associated with traditional doctor–patient interactions, although, in the present climate of health care reform, which is geared toward the cost-effective delivery of quality services, MMPs will become increasingly commonplace tools for patient education if they are demonstrated to be more effective than good clinical practice.
